# N-myc Downstream Regulated Gene 1 (NDRG1) Promotes Metastasis of Human Scirrhous Gastric Cancer Cells through Epithelial Mesenchymal Transition

**DOI:** 10.1371/journal.pone.0041312

**Published:** 2012-07-23

**Authors:** Hiroki Ureshino, Yuichi Murakami, Kosuke Watari, Hiroto Izumi, Akihiko Kawahara, Masayoshi Kage, Tokuzo Arao, Kazuto Nishio, Kazuyoshi Yanagihara, Hisafumi Kinoshita, Michihiko Kuwano, Mayumi Ono

**Affiliations:** 1 Department of Pharmaceutical Oncology, Graduate School of Pharmaceutical Sciences, Kyushu University, Fukuoka, Fukuoka, Japan; 2 Department of Molecular Biology, School of Medicine, University of Occupational and Environmental Health, Kitakyushu, Fukuoka, Japan; 3 Department of Diagnostic Pathology, Kurume University Hospital, Kurume, Fukuoka, Japan; 4 Department of Genome Biology, School of Medicine, Kinki University, Osakasayama, Osaka, Japan; 5 Division of Genetics, National Cancer Center Research Institute, Tokyo, Japan; 6 Department of Surgery, School of Medicine, Kurume University, Kurume, Fukuoka, Japan; 7 Laboratory of Molecular Cancer Biology, Department of Clinical Pharmaceutics, Graduate School of Pharmaceutical Sciences, Kyushu University, Fukuoka, Fukuoka, Japan; The University of Kansas Medical Center, United States of America

## Abstract

Our recent study demonstrated that higher expression of N-myc downregulated gene 1 (NDRG1) is closely correlated with poor prognosis in gastric cancer patients. In this study, we asked whether NDRG1 has pivotal roles in malignant progression including metastasis of gastric cancer cells. By gene expression microarray analysis expression of NDRG1 showed the higher increase among a total of 3691 up-regulated genes in a highly metastatic gastric cancer cell line (58As1) than their parental low metastatic counterpart (HSC-58). The highly metastatic cell lines showed decreased expression of E-cadherin, together with enhanced expression of vimentin and Snail. This decreased expression of E-cadherin was restored by Snail knockdown in highly metastatic cell lines. We next established stable NDRG1 knockdown cell lines (As1/Sic50 and As1/Sic54) from the highly metastatic cell line, and both of these cell lines showed enhanced expression of E-cadherin and decreased expression of vimentin and Snail. And also, E-cadherin promoter-driven luciferase activity was found to be increased by NDRG1 knockdown in the highly metastatic cell line. NDRG1 knockdown in gastric cancer cell showed suppressed invasion of cancer cells into surround tissues, suppressed metastasis to the peritoneum and decreased ascites accumulation in mice with significantly improved survival rates. This is the first study to demonstrate that NDRG1 plays its pivotal role in the malignant progression of gastric cancer through epithelial mesenchymal transition.

## Introduction

Gastric cancer is one of the most common malignancies in Japan and other Asian countries. The patient prognosis of scirrhous gastric carcinoma is particularly poor. Scirrhous gastric carcinoma is often accompanied by peritoneal dissemination and metastasis to the lymph nodes and liver, which are serious problems that have to be controlled. Gene expression profile revealed gene amplifications of K-sam and c-Met in 30–40% of scirrhous gastric cancers, and that the overexpression of various growth factors, such as transforming growth factor-β (TGF-β), platelet-derived growth factor (PDGF), insulin-like growth factor (IGF) and fibroblast growth factor-2 (FGF-2) [1]. Recent DNA microarray analysis demonstrated specific upregulation of several genes including *SPARC*, *RGEIII*, *S100A10*, and collagen type I [2]–[4].

Animal models that reflect the characteristics of cancerous peritonitis and metastasis to the peritoneum are important in understanding the development of malignant progression by gastric cancer. Yanagihara *et al.* [5] first established the HSC-58 cell line from human scirrhous gastric cancer, and then established two sublines, 58As1 and 58As9, with a high potential for peritoneal dissemination and lymph node metastasis by repeated orthotopic inoculation of HSC-58 cells into the gastric wall of athymic mice [6], [7]. These sublines often cause accumulation of ascites after orthotopic implantation and show increased expression of genes encoding proteases and proteins involved in cell adhesion, cell motility, proliferation and angiogenesis [6]. They also grow selectively in the gastric submucosa, and invade the deeper layer to reach the serosal plane of the stomach [7]. However, it remains unclear why they have acquired such a high potential for metastasis during selection.

In our present study, we used these highly metastatic cell lines to investigate how human gastric cancer cells could acquire metastatic potential. We first compared gene expression profiles between highly metastatic and low metastatic parental cell lines by microarray analysis. We focused on one gene named N-myc downstream regulated gene 1 (NDRG1) because it was found to be markedly upregulated in the highly metastatic gastric cancer cell lines compared to their counterpart cells. It was also previously reported that NDRG1 expression was a predicative marker for malignant progression and poor prognosis in gastric patients [8]. Consistent with this study, we observed that high NDRG1 expression was significantly correlated with tumor angiogenesis and malignant progression together with poor prognosis in gastric cancer [9]. We also reported that NDRG1 knockdown induces decreased production of potent angiogenic factors and tumor angiogenesis by lung cancer cells, and also that NDRG1 is a predictive marker for tumor angiogenesis and poor prognosis in patients with no-small cell lung cancer [10]. NDRG1, one of the four NDRG family genes, thus shows diverse functions [11], and NDRG1 functions either as metastasis suppressor or as oncogenic and malignant promoter, depending on tumor types [11], [12]. Furthermore, expression of NDRG1 gene is closely controlled by N-Myc and related Myc family proteins [13] and overexpression of c-Myc induced epithelial mesenchymal transition (EMT) in mammary epithelial cells [14]. The important role of EMT is often referred in its close context of development and tumor progression including cancer metastasis [15], [16]. However in these studies, the regulatory role of NDRG1 was not studied. In our present study, we investigated the metastatic potential of gastric cancer cells by its correlation with EMT-based role of NDRG1.

## Results

### Comparison of Cell Growth, Cell Morphology, Tumor Growth, Dissemination and Gene Expression Profiles between Highly Metastatic Gastric Cancer Cell Lines and their Low Metastatic Counterpart

**Figure 1:**
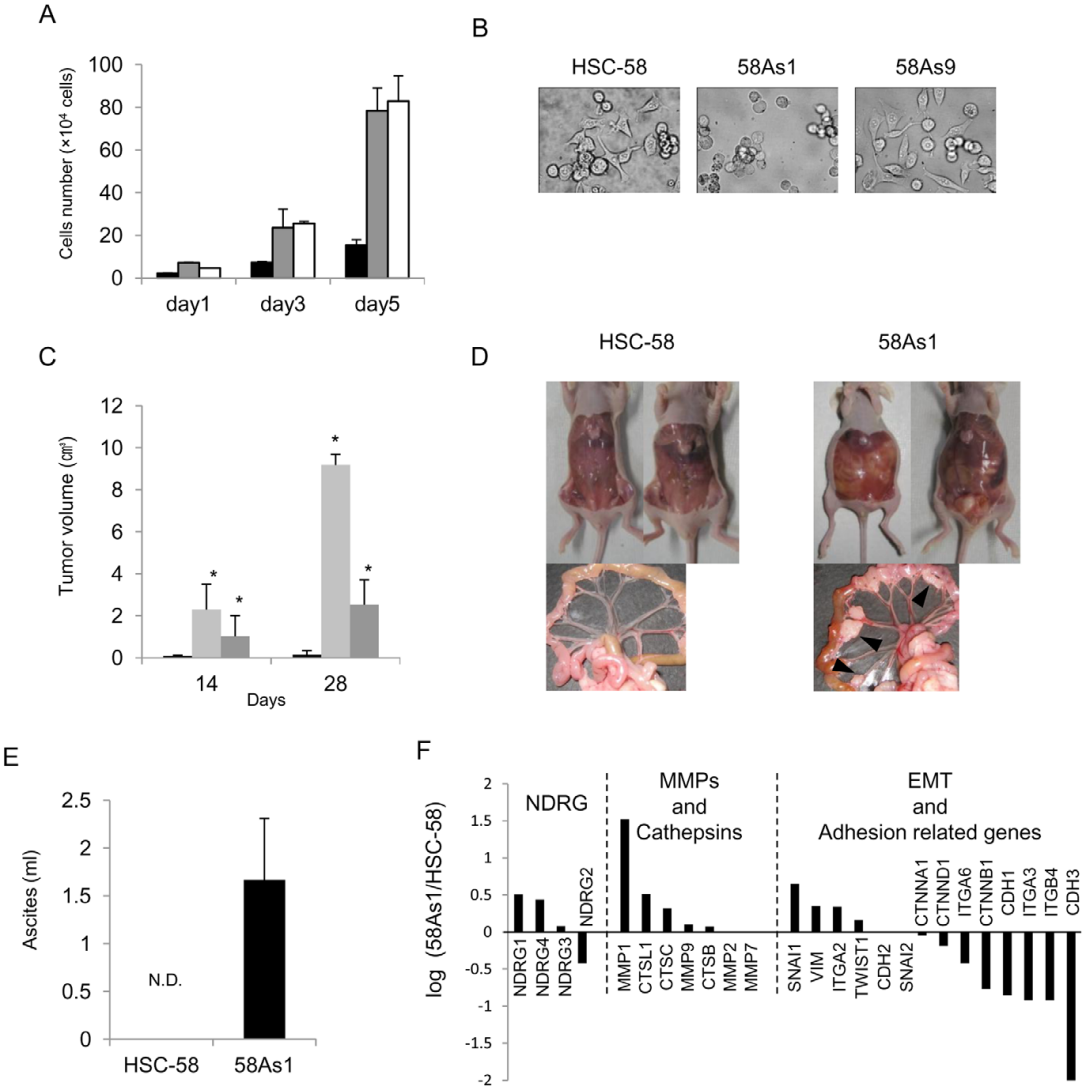
Biological properties of low and highly metastatic gastric cancer cell lines *in vitro* and *in vivo*. (A) Comparison of cell proliferation rates *in vitro*. Cells were seeded on day 0 (5×10^4^ cells/dish) and proliferation was measured in RPMI 1640 containing 10% FBS. Doubling times were 34, 27, and 23 hr for HSC-58 (black), 58As1 (gray) and 58As9 cells (white), respectively. (B) Morphology of gastric cancer cell line *in vitro*. HSC-58 and 58As9 cell growth was visible as attached layers with fibroblastic morphology. 58As1 cells showed suspension-type cell growth with round morphology. (C) Tumor growth of gastric cancer cell lines at day 14 and 28. HSC-58 (black), 58As1 (light gray), and 58As9 (dark gray) were subcutaneously implanted with 1×10^7^ cells at day 0. 58As1 or 58As9 cells showed significantly (*p<0.01) higher tumor growth rates than HSC-58 cells. (D, E) Peritoneal dissemination and ascites formation *in vivo*. Typical figures show high and low peritoneal dissemination and ascites accumulation at day 55 after inoculation of 1×10^6^ cells into peritoneal cavity. Each mouse showed ascites accumulation of 0.7–2.5 ml and 5–12 nodules on the mesenterium following 58As1 inoculation, but this was not apparent with HSC-58 cells. N.D., not detectable. (F) Microarray analysis on expression of genes that are up- or down- regulated in highly metastatic cell line, 58Asl, as compared with HSC-58. Relative expression rates are presented on genes belonging to three biological functions.

Consistent with previous studies [6], [7], the highly metastatic cancer cell lines 58As1 and 58As9 showed higher growth rates than their parental counterpart, HSC-58, with doubling times of 23–26 hr compared with 30 hr for parental cells (Figure 1A). 58As1 cells were easily detached and showed suspension-type cell growth with round morphology, while HSC-58 and 58As9 cells were attached and showed layered cell growth with a fibroblastic morphology (Figure 1B). Both 58As1 and 58As9 cells showed much higher tumor growth rates in a subcutaneous xenograft model compared to parental HSC-58 cells (Figure 1C). Previous studies demonstrated that the orthotopic transplantation of 58As1 cells induced much higher peritoneal dissemination and ascites accumulation than that of HSC-58 cells [6], [7]. We further confirmed the peritoneal dissemination of the cells by implanting them into the peritoneal cavity of mice. Mice implanted with 58As1 cells showed the appearance of an average of 8±3 visible nodules in the mesenterium of each mouse and accumulation of ascites (ranging from 0.7–2.5 ml/mouse) (Figure 1D, E). By contrast, mice implanted with parental HSC-58 cells showed the formation of few if any tiny nodules, and no apparent accumulation of ascites.

We next compared gene expression profiles between 58As1 cells and their parental counterpart, HSC-58, using DNA microarray analysis. Of the 41,000 RNA transcripts and variants, we identified 3691 differentially expressed genes that were upregulated to 2-fold, and 3536 genes that were downregulated to 0.5-fold or less in 58As1 cells compared to HSC-58 cells. Figure 1F presented genes that were differentially expressed in 58As1 cells compared to HSC-58 cells, limited to those belonging to the NDRG family, and genes related to metastasis, including matrix metalloproteinases (MMPs)/cathepsins, adhesion and epithelial-mesenchymal-transition (EMT). Of the four genes in the NDRG family [17], the expression of NDRG1 and NDRG4 was upregulated in the highly metastatic cell line (58As1) compared to the parental cell line (HSC-58). The expression of EMT-related genes in 58As1 cells was especially affected by the acquisition of a high metastatic potential. The expression of epithelium-specific genes, including E-cadherin (*CDH1*), P-cadherin (*CDH3*) and β-catenin (*CTNNB1*), was downregulated. By contrast, only MMP1 (*MMP1*) expression was markedly upregulated; the expression of cathepsin L (*CTSL1*) was increased, but that of cathepsin B (*CTSB*) was not. The expression of mesenchyme-specific genes, vimentin (*VIM*) and Snail (*SNAI1*), a key transcription factor for the suppression of E-cadherin expression, was upregulated.

### Enhanced NDRG1 Gene Expression in Highly Metastatic Gastric Cancer Cell Lines

**Figure 2:**
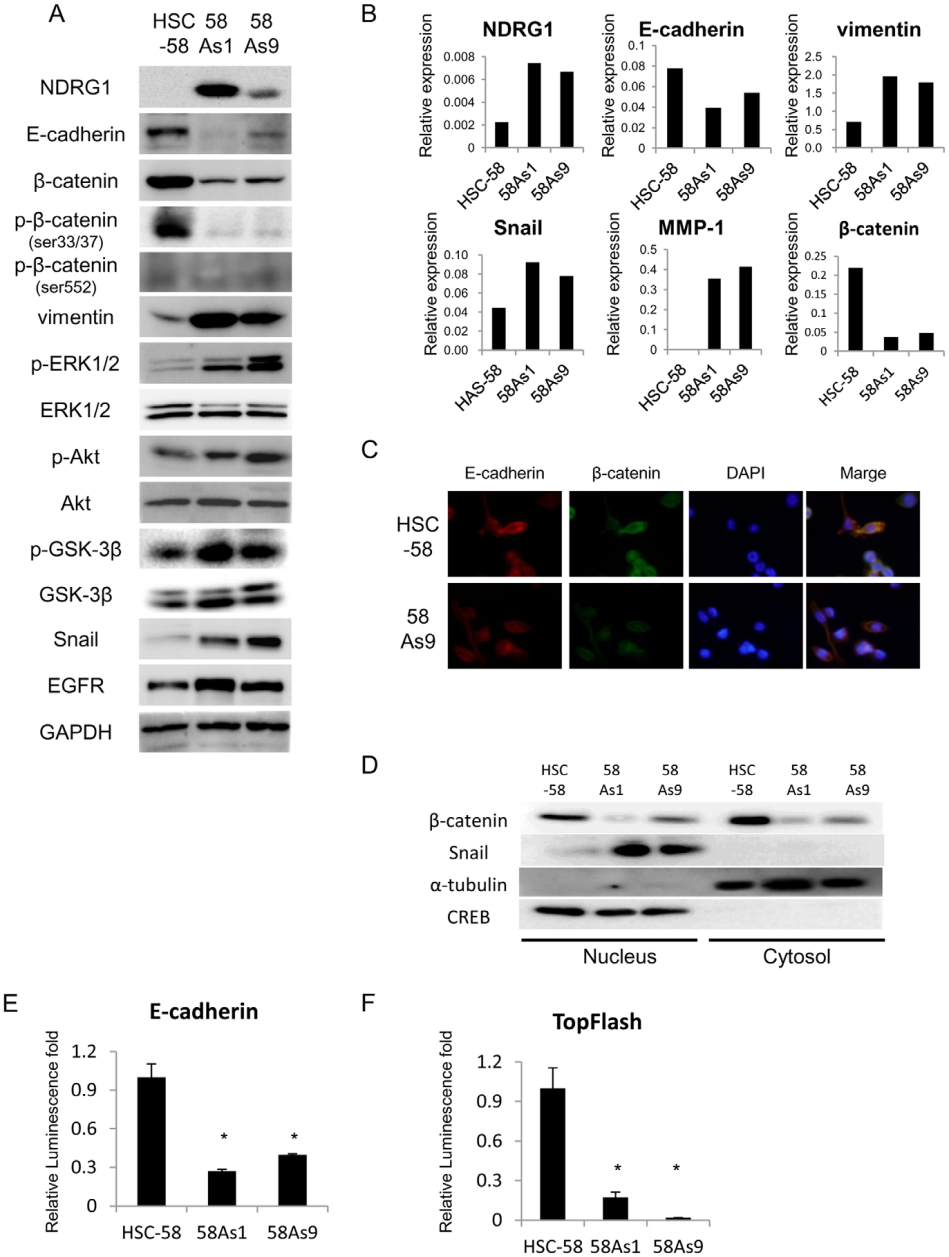
Comparison of protein and mRNA expression levels of various factors between low and highly metastatic gastric cancer cell lines. (A) Western blot analysis of total cell lysates shows protein expression levels of NDRG1, growth factor receptor, EMT-related proteins, Wnt/β-catenin-related proteins, and other factors in HSC-58, 58As1 and 58As9 cells. (B) Comparison of mRNA expression levels of NDRG1, E-cadherin, vimentin, Snail, MMP-1 and β-catenin in HSC-58, 58As1 and 58As9 cells by qRT-PCR analysis. (C) Immunocytochemical analysis of E-cadherin and β-catenin in HSC-58 and 58As9, using specific antibodies against E-cadherin, β-catenin and DAP1. Magnification×200. (D) Western blot analysis shows expression of β-catenin and Snail in nucleus and cytosol fraction. CREB, a nuclear marker, and α-tubulin, a cytosol marker. (E,F) Comparison of luciferase activity driven by E-cadhrin promoter and β-catenin (TopFlash) driven promoter between HSC-58 and its highly metastatic cell lines. The relative promoter activity is presented when normalized by the activity in HSC-58. *p<0.01.

We further compared the protein expression of several genes in HSC-58, 58As1 and 58As9 cells (Figure 2A). The expression of NDRG1 was markedly augmented, and that of vimentin, Snail, p-ERK1/2, p-Akt, and p-GSK-3β was also increased, in both 58As1 and 58As9 cells compared with HSC-58 cells. There was no apparent expression of Wnt3a and Wnt5a in these cell lines (data not shown). By contrast, we observed decreased expression of E-cadherin and β-catenin in 58As1 and 58As9 cells compared with HSC-58 cells (Figure 2A). Phosphorylation of β-catenin Ser33/37 and Ser552 was found to be much less in 58As1 and 58As9 than HSC58. Consistent with the protein expression levels, mRNA expression levels of NDRG1, vimentin, Snail and MMP-1 were higher in both highly metastatic cell lines than their parental counterpart (Figure 2B). There was much less mRNA expression of E-cadherin and β-catenin in both 58As1 and 58As9 than HSC-58.

Immunocytochemical analysis revealed that localization of E-cadherin both in cytoplasm and membrane, and β-catenin was mostly localized in cytoplasm in both HSC-58 and 58As9 cells (Figure 2C). Immunocytochemical analysis for 58As1 could not be performed as 58As1 grows in suspension. Furthermore, expression of β-catenin was relatively lower in both cytoplasm and nucleus but that of Snail was found to be much higher in nucleus of 58As1 and 58As9 than HSC-58 (Figure 2D). We also examined E-cadherin promoter activity (Figure 2E) and β-catenin induced reporter activity by TopFlash reporter assay (Figure 2F) and found that there was significant decrease in the luciferase activity driven by E-cadherin promoter and also by β-catenin in both 58As1 and 58As9 compared with HSC-58, consistent with mRNA levels of both genes.

### NDRG1 Knockdown Induces the Upregulation of E-cadherin and the Downregulation of Vimentin and Snail in Highly Metastatic Cells

**Figure 3:**
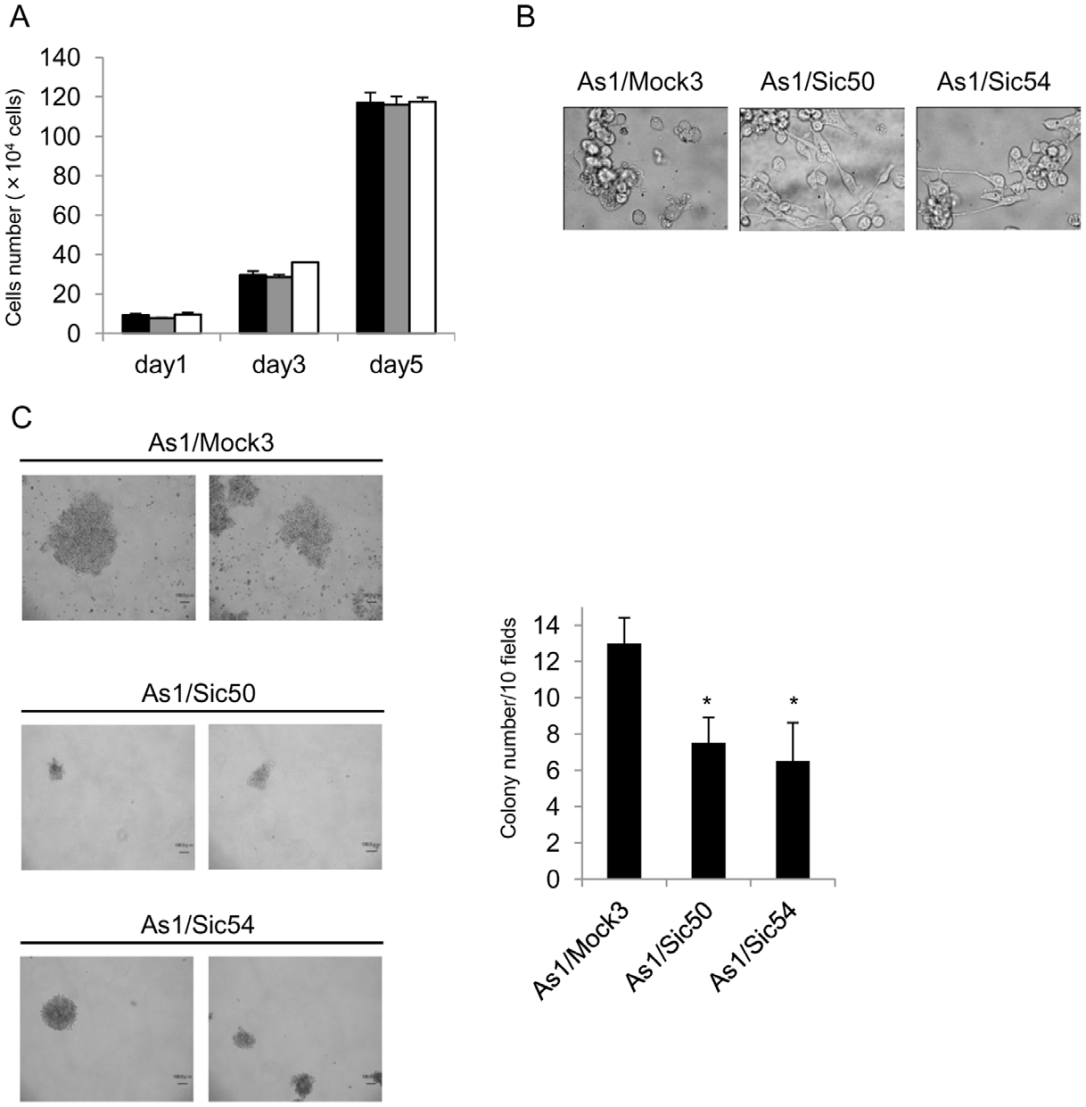
Comparison of biological characteristics between highly metastatic 58As1 and its NDRG1 knockdown cell lines. (A) Cell proliferation in RPMI 1640 containing 10% FBS was followed after seeding 5×10^4^ cells/dish on day 0. Doubling times were similar for As1/Mock3(black), As1/Sic50(gray) and As1/Sic54(white) cells (26–30 hr). (B) Cell morphology of As1/Mock3, As1/Sic50 and As1/Sic54. Typical cell morphology in culture shows As1/Mock3 suspension-type cell growth and attached layer-type cell growth of both NDRG1 knockdowned cell lines. (C) Representative images of colonies of As1/Mock3, As1/Sic50 and As1/Sic54 when incubated for 14 days in soft agar (left panel). Quantitative analysis of colony formation activity by three cell lines when 5 dishes for each line were scored (right panel). Significant differences (*p<0.01) in colony number between 58As1 and its NDRG1 knockdown cell lines.

We next examined whether NDRG1 knockdown specifically affected the expression of EMT-related genes in the highly metastatic cell line, 58As1. We established two stable NDRG1 knockdown cell clones, As1/Sic50 and As1/Sic54 by transfecting NDRG1 shRNA into 58As1 cells. Both As1/Sic50 and As1/Sic54 cells showed similar growth rates to each other and their parental counterpart As1/Mock3, with doubling times of 25–27 hr in culture (Figure 3A). Both NDRG1 silenced cell lines showed tight adhesion of cells to the culture dish and fibroblastic morphology, whereas As1/Mock3 cells showed a suspension-type cell growth with round cell morphology (Figure 3B). We next examined the effect of NDRG1 silencing on the tumorigenic activity of 58As1 cells using an anchorage-independent growth assay. Both NDRG1 silenced cell lines showed significant reduction of their anchorage-independent growth, as indicated by colony number in soft agar (^*^ *p<*0.01) (Figure 3C).

**Figure 4:**
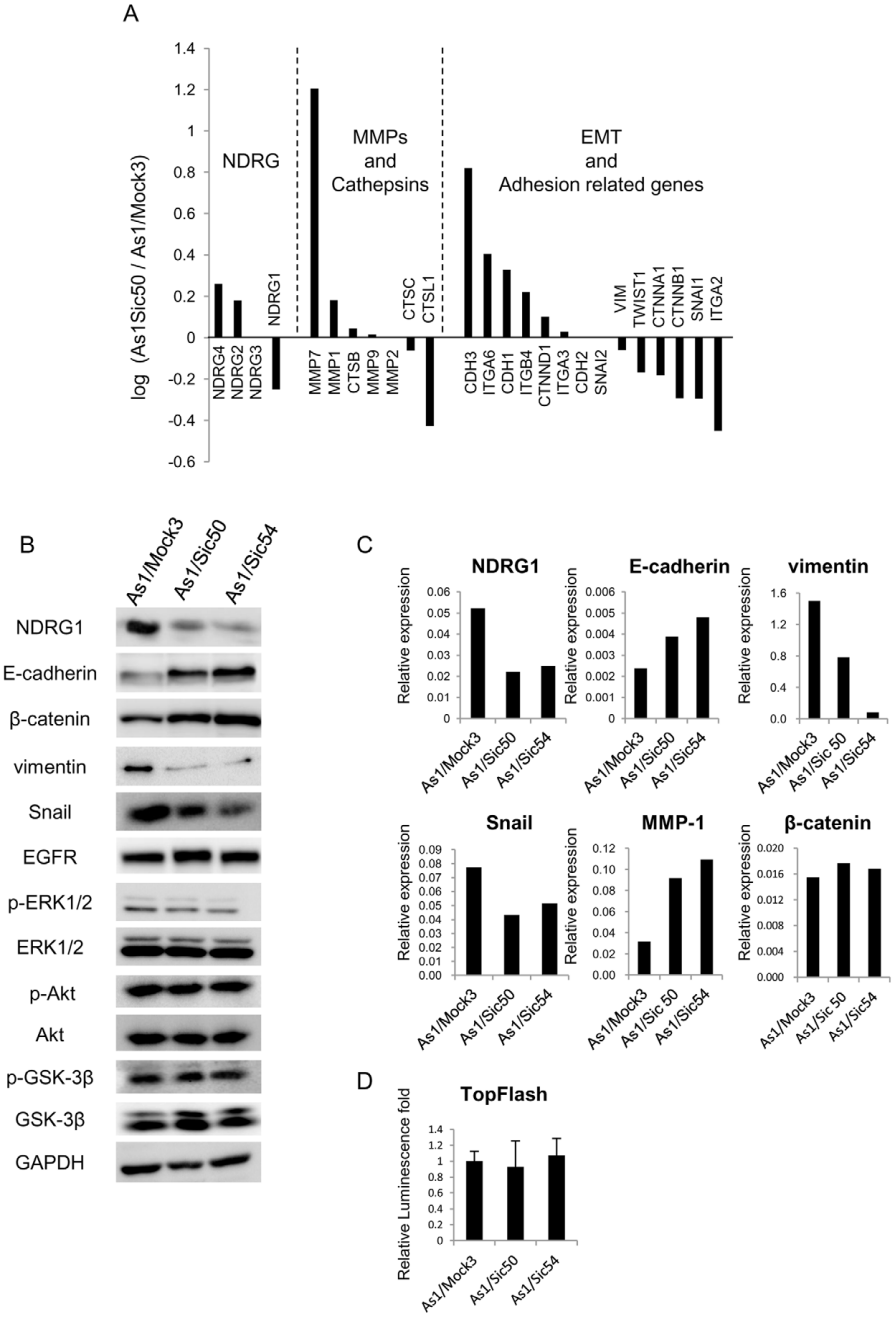
Altered expression of EMT-related factors by NDRG1 knockdown in highly metastatic 58As1. (A) Microarray analysis for the effect of NDRG1 knockdown on expression of genes that are up- or down- regulated in Asl/Sic50 versus As1/Mock3. Relative expression rates are presented on genes belonging to three biological functions. (B) Comparison of protein expression levels of NDRG1, EMT-related proteins, β-catenin, Akt, p-Akt, ERK1/2, p-ERK1/2, GSK-3β, p-GSK-3β and EGFR by western blot analysis with total cell lysate. (C) The mRNA expression of NDRG1, E-cadherin, vimentin, Snail, MMP-1 and β-catenin was determined by qRT-PCR analysis. (D) Comparison of luciferase activity driven by β-catenin (TopFlash) between As1/Mock3 and its NDRG1 knockdowned cell lines. Relative luminescence fold is presented when normalized by the value in As1/Mock3. Each column is average of triplicate trials±SD.

Then we compared the gene expression profiles between As1/Mock3 and As1/Sic50 cells using DNA microarray analysis (Figure 4A). NDRG1 knockdown resulted in increased expression of the E-cadherin (*CDH1*) and P-cadherin (*CDH3*) but decreased expression of the vimentin (*VIM*) and Snail (*SNAI1*). We next performed western blot and qRT-RCR analyses for several molecules that were modulated by NDRG1 knockdown in the microarray analysis (Figure 4B, C). These two cell lines showed much lower expression of both NDRG1 mRNA and protein compared to the As1/Mock3 cells. Enhanced expression of E-cadherin was consistently observed in As1/Sic50 and As1/Sic54 cells compared to As1/Mock3 cells by western blot analysis. In As1/Sic50 and As1/Sic54 cells, both western blot and qRT-PCR analyses confirmed decreased expression of vimentin and Snail without affecting expression of p-ERK1/2, ERK1/2, p-Akt, Akt, p-GSK-3β and GSK-3β (Figure 4B, C).

On the other hand, NDRG1 is well known to suppress metastasis and cell proliferation by prostate and colon cancer cells. Recent studies have demonstrated that NDRG1 modulates Wnt-β-catenin signaling pathway with enhanced expression of E-cadherin in human prostate and colon cancer cell [18], [19]. However, NDRG1 knockdown in 58As1 cells did not affect the β-catenin expression both at protein and mRNA level (Figure 4B, C). And also β-catenin driven promoter activity by TopFlash reporter assay revealed no difference in the promoter activity between As1/Mock and NDRG1 silenced cell lines. Therefore, β-catenin expression was not affected by NDRG1 knockdown. NDRG1 knockdown thus consistently increased expression of E-cadherin and decreased expression of Snail and vimentin in the highly metastatic cell line. However, there was no apparent change in expression of β-catenin by NDRG1 knockdown.

### Enhanced E-cadherin Promoter Activity by NDRG1 Knockdown through Snail in Gastric Cancer Cells

**Figure 5:**
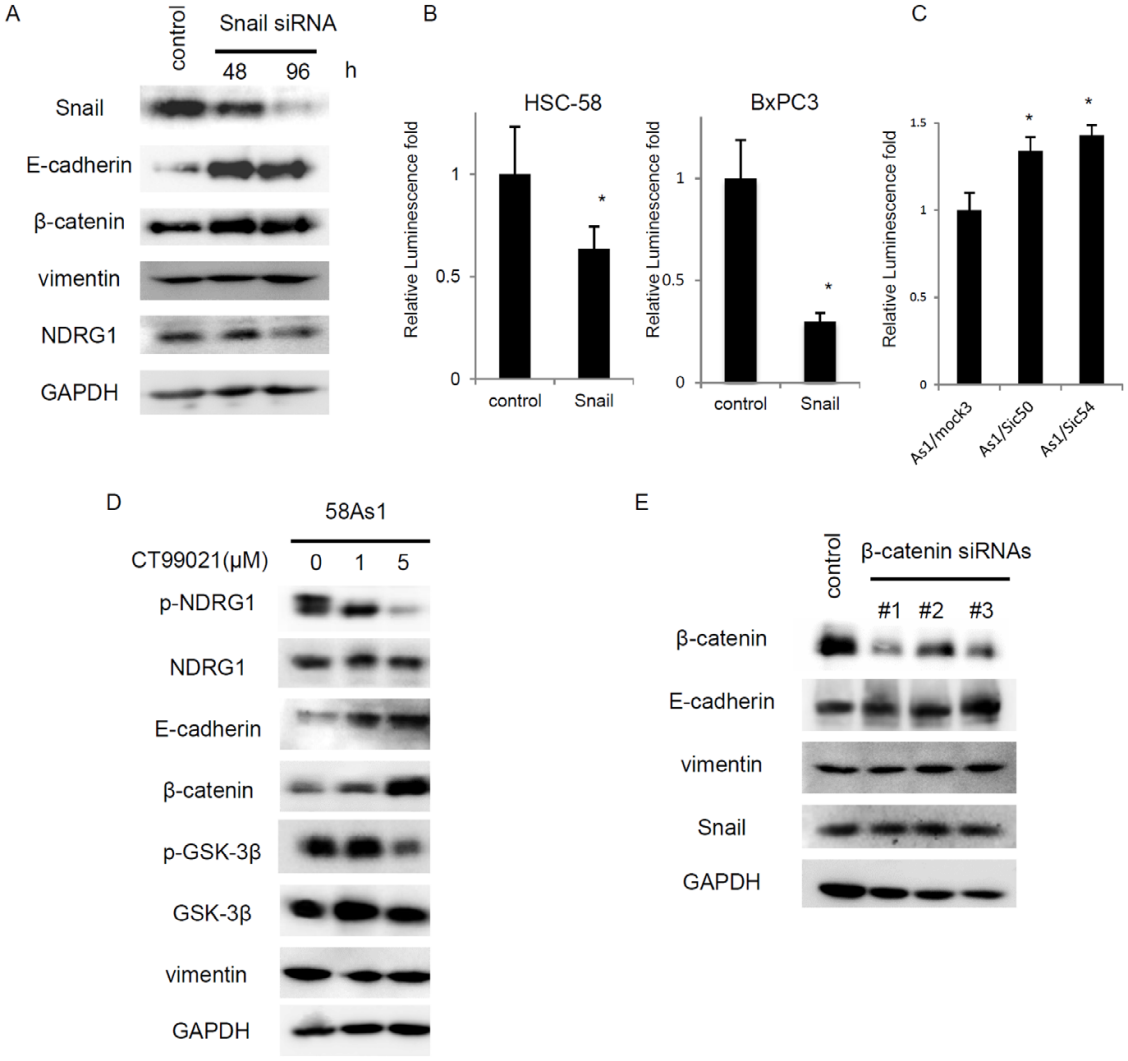
Enhancement of E-cadherin promoter activity by NDRG1 knockdown in highly metastatic gastric cancer cell. (A) Comparison of protein expression levels of E-cadherin, β-catenin, NDRG1 and vimentin when transiently treated with Snail siRNA for 0, 48 and 96 hr by western blot analysis in HSC-58 cell. (B) E-cadherin promoter-driven luciferase activity in the absence or presence of Snail expression in HSC-58 and BxPC-3 cells. E-cadherin-luc was transfected with or without pcDNA3-Snail, and the luciferase activity was measured. Each column is average of triplicate trials (*p<0.05). (C) Comparison of E-cadherin promoter-driven luciferase activity (E-cadherin-luc) in As1/Mock3, As1/Sic50 and As1/Sic54. Each column is average of triplicate trials (*p<0.05). (D) The effect of CT99021 on protein expression of NDRG1 and various EMT-related molecules by Western blot analysis. 58Asl cells were treated with indicated doses of the drug for 24 hr. (E) The effect of β-catenin knockdown by its siRNAs on expression of E-cadherin. HSC-58 cells were transfected with siRNAs for 24 hr, and total cell lysates were analyzed by Western blot analysis.

Of various transcription factors that suppress expression of E-cadherin including Snail, Slug, Twist, TCF4 and SIP1 [20], expression of Snail was specifically modulated by NDRG1 in gastric cancer cells. We examined whether expression of E-cadherin and/or vimentin could be regulated by Snail in HSC-58 cells. Transient treatment with Snail siRNA resulted in increased expression of E-cadherin, but not that of vimentin and NDRG1, in time-dependent manner (Figure 5A). There was only a slight if any increase in β-catenin expression by Snail knockdown. Furthermore, E-cadherin promoter-driven luciferase activity was significantly suppressed by transfection of Snail complementary DNA in two cancer cell lines tested (Figure 5B). We next compared E-cadherin promoter activity between As1/Mock3 and its NDRG1 silenced cell lines. As seen in Figure 5C, there was significant (*p<0.05) enhancement of E-cadherin promoter activity by NDRG1 knockdown.

GSK-3β is a key enzyme for activation of EMT pathway [20], [21], and also for phosphorylation of NDRG1 [22], [23]. Expression of p-GSK-3β was increased in the highly metastatic cell line, 58As1 (see Figure 2A). We examined whether GSK-3β was involved in NDRG1 induced altered expression of E-cadherin and vimentin. Treatment with an inhibitor (CT99021) of GSK-3β resulted in decreased expression of p-NDRG1 and p-GSK-3β, and also increased expression of E-cadherin and β-catenin in 58As1 cells (Figure 5D). As shown in Figure 5E, β-catenin knockdown did not affect expression of E-cadherin, vimentin and Snail.

### NDRG1 Knockdown Induces Mesenchymal Epithelial Transition and Suppresses Metastasis to the Peritoneum by Highly Metastatic Gastric Cancer Cells

**Figure 6:**
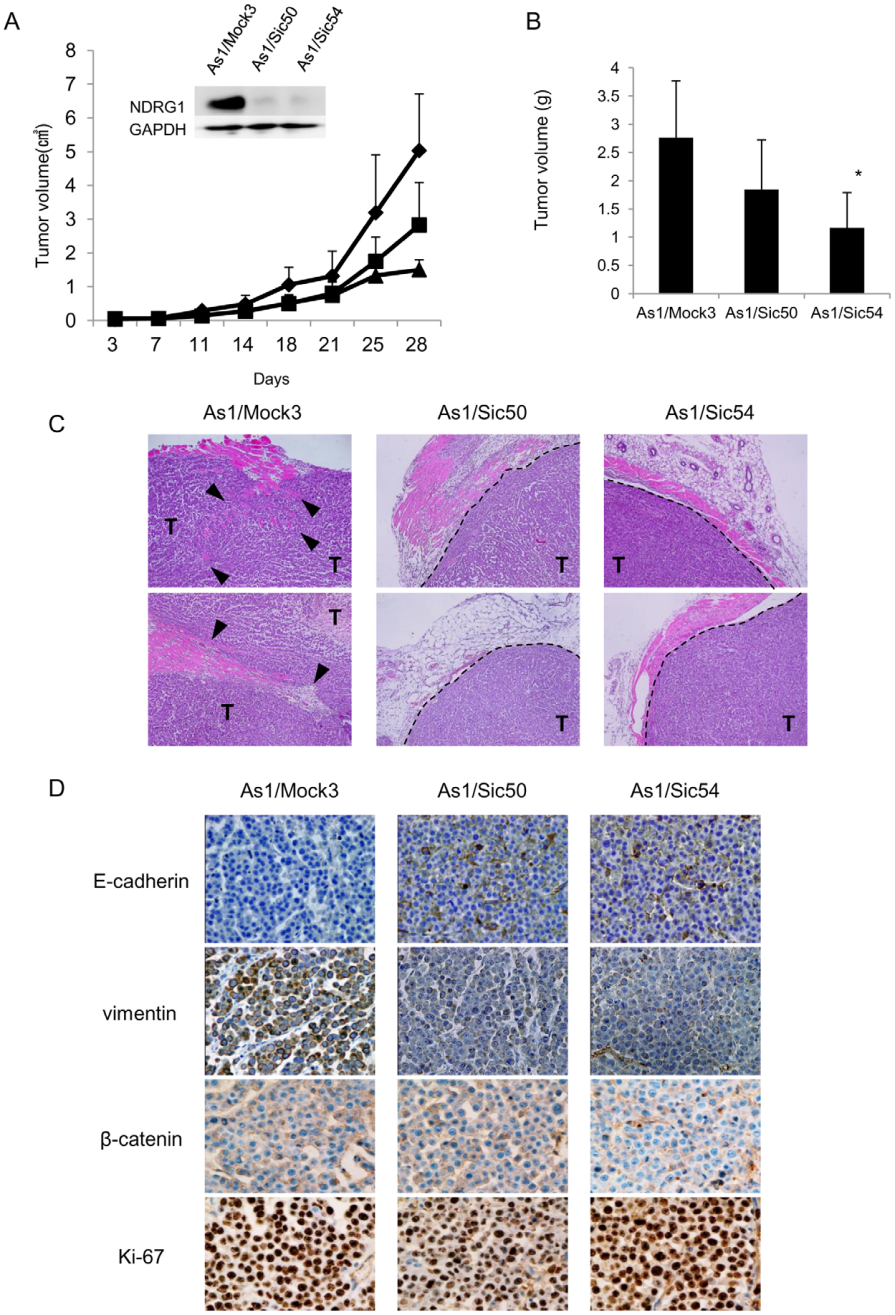
NDRG1 knockdown suppresses tumor growth and invasion by highly metastatic cancer cell. (A) Tumor growth was followed after subcutaneous inoculation of 5×10^6^ As1/Mock3 (♦), As1/Sic50 (▪) and As1/Sic54 cells (▴). Inlet shows no apparent NDRG1 expression in either As1/Sic50 or As1/Sic54 tumors. (B)Tumor volume for As1/Sic50 (*p* = 0.87) and As1/Sic54 cells were slightly smaller than for As1/Mock3 (day28). Each column is an average of four animals (±SD) (*p<0.05). (C) Representative H&E staining of tumor samples. Dashed lines indicate tumor margins, closed triangles indicate invasion of cancer cell in normal tissue. ‘T’ indicates tumor cells. (D) IHC images of E-cadherin, vimentin, β-catenin and Ki-67 expression in As1/Mock3, As1/Sic50 and As1/Sic54 tumors.

Comparing tumor growth rates of As1/Mock3, As1/Sic50 and As1/Sic54 cells in a xenograft model revealed slower tumor growth rates of both As1/Sic50 and As1/Sic54 by NDRG1 knockdown (Figure 6A, B). NDRG1 knockdown suppressed the local invasion of As1/Mock3 tumor cells into the surrounding stroma and adjacent adipose and/or muscle tissue, and encapsulated tumor growth (Figure 6C). High expression of E-cadherin was observed in As1/Sic50 and As1/Sic54 tumors, while high expression of vimentin was observed in cancer cells of As1/Mock3 tumors (Figure 6D). By contrast, there was almost no change in expression of β-catenin in As1/Sic50 and As1/Sic54 tumors as compared with As1/Mock3 tumor.

**Figure 7:**
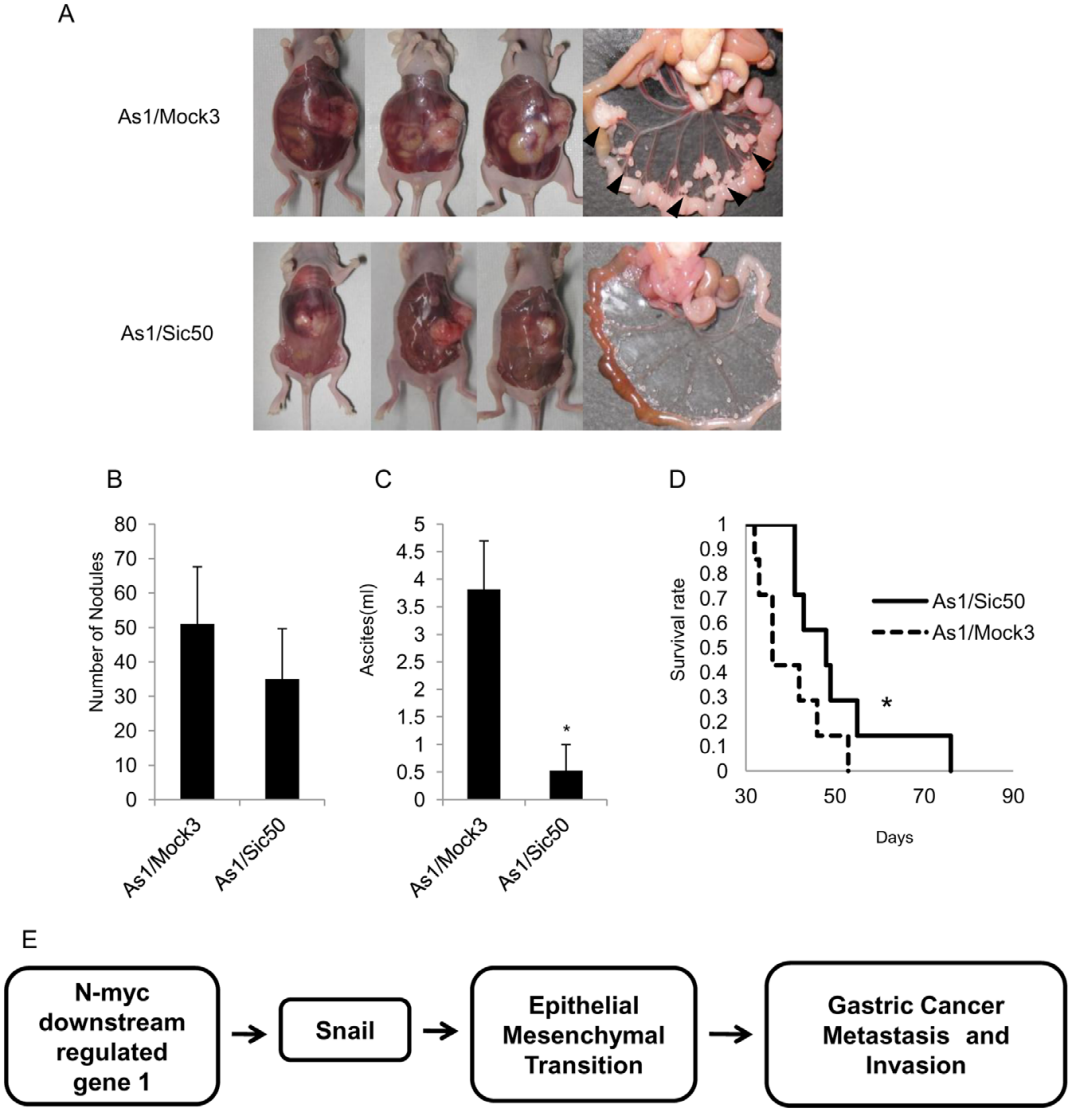
Suppression of peritoneal dissemination by NDRG1 knockdown. (A) Macroscopic images show enlarged peritoneal cavity and metastatic nodules by As1/Mock3 and As1/Sic50. Arrowheads show nodules. (B) Number of metastatic nodules in the mesenterium was 51±16 (As1/Mock3) and 35±14 (As1/Sic50) (*p* = 0.21), but the As1/Mock3 nodule size was 3–4 times larger than those of As1/Sic50. (C) Comparison of the volume of ascites between As1/Mock3 (3.9±1.0 ml) and As1/Sic50 cells (0.5±0.6 ml) following orthotopic implantation (n = 7) (* *p*<0.01). (D) Survival curves show that survival rate in As1/Sic50 tumor-bearing mice was significantly (* *p*<0.01) longer than that of As1/Mock3 tumor-bearing mice (n = 6). (E) Our hypothetic model how NDRG1 overexpression promotes metastasis including peritoneal dissemination through alteration of EMT by scirrhous gastric cancer cells, possibly through modification of Snail expression.

To examine whether NDRG1 knockdown could affect metastasis to the peritoneum and accumulation of ascites, we compared the metastatic potential of As1/Sic50 and As1/Mock3 cells after orthotopic transplantation. Figure 7A shows representative images of the enlarged abdominal cavity and the presence of cancer nodules in the peritoneum. After orthotopic inoculation of As1/Sic50 cells, the number of the nodules appearing in the peritoneum was about 30% less than those resulting from As1/Mock3 cells, but this decrease was not statistically significant (*p* = 0.21) (Figure 7B). However nodules resulting from As1/Sic50 cells showed much smaller sizes than those from As1/Mock3 cells (Figure 7A). The accumulation of ascites by As1/Sic50 cells was significantly (*p<0.01) decreased, to about 10% of that induced by As1/Mock3 cells (Figure 7C). Furthermore, there was a significant (*p<0.01) increase in the survival rate of mice inoculated with As1/Sic50 cells (51±16 days) compared with 35±14 days for mice inoculated with As1/Mock3 cells, indicating that NDRG1 knockdown prolongs survival (Figure 7D).

## Discussion

We have previously reported that NDRG1 is closely correlated with tumor angiogenesis and poor survival in patients with gastric cancer, suggesting that NDRG1 is a predictive biomarker for malignant progression of gastric cancer [9]. From our present study, we observed the following properties underlying the acquisition of a high metastatic potential in gastric cancer: microarray, western blot and RT-PCR analyses together revealed upregulation of NDRG1 in the highly metastatic cell lines, 58As1 and 58As9, compared with their low metastatic parental counterpart, HSC-58; higher expression of vimentin, Snail, and MMP-1, and lower expression of E-cadherin and β-catenin were evident in the highly metastatic cell lines compared with their parental counterpart; of the genes downregulated in the highly metastatic cell line, NDRG1 knockdown resulted in upregulation of E-cadherin, and downregulation of vimentin and Snail, but almost no effect on expression of β-catenin; E-cadherin promoter activity was significantly augmented by NDRG1 knockdown; NDRG1 knockdown also suppressed peritoneal dissemination and accumulation of ascites, and invasion of highly metastatic cells into surrounding normal tissues.

In the present study, NDRG1 knockdown resulted in the suppression of metastasis by highly metastatic gastric cancer cells (Figure 6, 7), suggesting that NDRG1 may be a metastasis promoter gene rather than a metastasis suppressor gene in gastric cancer. Our present study strongly supports the idea that whether NDRG1 promotes or suppresses the malignant progression of human cancer depends upon tumor types and/or differentiation status [11], [12]. However, it remains unclear why NDRG1 functions as tumor suppressor or promoter depending upon tumor types or histological types. Our previous study demonstrated that NDRG1 suppresses tumor growth and angiogenesis of pancreas cancer through NDRG1 driven attenuation of NF-κB signaling pathway [24], [25]. Recent study has reported that expression of metastasis suppressor gene, *KAI1* gene, is involved in NDRG1 mediated metastasis suppression of prostate cancer through ATF3-NF-κB pathway [26]. Further study is required to understand which regulatory mechanism is specifically responsible for NDRG1 driven promotion of malignant progression by gastric cancer cells.

EMT is a recent highlight that could be closely associated with cancer malignant progression including acquirement of highly metastatic potential [15], [16]. In our present study, NDRG1 knockdown enhanced the expression of E-cadherin and suppressed the expression of vimentin both *in vitro* and *in vivo*. NDRG1 may play a key role in the switch from a polarized epithelial phenotype to a highly motile mesenchymal phenotype. Partial or complete loss of E-cadherin is often observed during the progression toward malignancy in numerous tumors, including gastric cancer [27], [28]. Oliveira et al. [29] reported a close link between E-cadherin loss and high metastatic potential in gastric cancer. Chan et al. [30] also reported soluble E-cadherin as a biomarker for prolonged survival of gastric cancer patients. NDRG1 knockdown resulted in relatively higher E-cadherin expression in As1/Sic50 tumors than in As1/Mock3 tumors, suggesting that NDRG1 may specifically control the EMT possibly through the transcription factor Snail by gastric cancer cells (Figure 7E).

Wnt signaling has pivotal roles in the malignant progression and metastasis by lung and breast cancer cells [31], [32], and Wnt signaling also induces EMT which involves the metastatic progression of epithelial cancer [31], [33]. NDRG1 is known as a metastasis suppressor gene in prostate and colorectal cancer cell [11], [12]. Liu et al [18] has reported that NDRG1 suppresses metastasis through Wnt/β-catenin signaling pathway in prostate cancer cells. A relevant study by Chen et al [19] has also demonstrated that NDRG1 modulates TGF-β-induced EMT through altered expression of β-catenin and E-cadherin in colorectal cancer cell. Our present study showed decreased expression of β-catenin in both highly metastatic cell lines compared to the parental counterpart, HSC-58. However, both western blot and qRT-PCR analyses showed no marked change in expression levels of β-catenin and also β-catenin driven promoter activity between highly metastatic 58As1 cells and its two NDRG1 silenced cell lines. It seems less likely that β-catenin plays a pivotal role in suppression of metastasis by NDRG1 knockdown in highly metastatic cancer cells.

On the other hand, GSK-3β is also known to play a role in the control of EMT [20], [21], and GSK-3β is an essential enzyme for the phosphorylation of NDRG1, an excellent substate of GSK-3β [22], [23]. The expression of p-GSK3β was relatively higher in the highly metastatic cell lines than their parental cell line (Figure 2B). Treatment with a potent inhibitor of GSK-3β resulted in a suppressed expression p-NDRG1, accompanied by upregulation of E-cadherin and β-catenin (Figure 5D). However, there was almost no difference in expression of GSK-3β and p-GSK-3β by NDRG1 knockdown in the highly metastatic cells (Figure 4B), suggesting that GSK-3β may not play a major role in the induction of EMT through NDRG1 in gastric cancer cells.

Snail is a representative transcription factor that controls expression of E-cadherin [20]. Of various regulatory factors that transcriptionally control E-cadherin, expression of Snail was specifically modulated by NDRG1 in gastric cancer cell lines used in this study. Snail knockdown induced upregulation of E-cadherin (Figure 5A), and exogenous tarnsfection of Snail cDNA suppressed E-cadherin promoter activity (Figure 5B).

In highly metastatic gastric cancer cell lines, expression of Snail was upregulated as compared to their low metastatic counterpart, HSC-58 (Figure 2A, B). Both NDRG1 silenced cell lines, As1/Sic50 and As1/Sic54, showed decreased expression of Snail as compared to their highly metastatic counterpart, As1/Mock3 (Figure 4B, C). Furthermore, E-cadherin promoter activity was significantly stimulated in both NDRG1 silenced cell lines as compared to their highly metastatic counterpart (Figure 5C).

In conclusion, NDRG1 knockdown induced the upregulation of E-cadherin and downregulation of vimentin and Snail, and suppressed the invasion, metastasis and accumulation of ascites by the highly metastatic gastric cancer cells. This NDRG1 mediated regulation of E-cadherin and/or vimentin expression affected epithelial mesenchymal transition of gastric cancer cells. This NDRG1 induced modification of EMT seems to be at least in part attributable to the transcriptional factor Snail. NDRG1, in its close context with EMT-related genes, might participate in the acquisition of a high metastatic potential by progressive gastric cancer cells.

## Materials and Methods

### Materials and Cell Lines

HSC-58 was established from human scirrhous gastric carcinomas and 58As1 and 58As9 were independently established by repeated orthotopic implantation of HSC-58 cells into the gastric wall of athymic mice as described previously [5], [6]. As1/Sic50 and As1/Sic54 cells were established by the stable transfection of NDRG1 shRNA into 58As1 cells. All cell lines were maintained in RPMI-1640 medium supplemented with 10% fetal bovine serum (FBS). BxPC-3, a human pancreatic cancer cell lines, was obtained from the American Type Culture Collection (Manassas, VA). Anti-NDRG1 antibody was generated as previously described [34]. Other antibodies were purchased as follows: anti-β-actin antibody and anti-Snail antibody were from Abcam; anti β-catenin antibody, anti-β-catenin (ser 33/37), anti-β-catenin (ser 552), anti-Wnt, anti-Ki-67, anti-GSK-3β antibody, anti-EGFR antibody, anti-GAPDH antibody, anti-p-GSK-3β antibody, anti-ERK1/2 antibody anti-p-ERK1/2 antibody, anti-AKT antibody, anti-p-AKT antibody were from Cell Signaling Technology; anti-vimentin antibody was from Calbiochem; anti-E-cadherin antibody was from BD. CT99021 was purchased from Axon Medchem BV (Netherlands).

### Gene Expression Microarrays

Complementary RNA was amplified, labeled, and hybridized to a 44K Agilent 60-mer oligomicroarray according to the manufacturer’s instructions (Agilent Technologies). All hybridized microarray slides were scanned by an Agilent scanner. Relative hybridization intensities and background hybridization values were calculated using Agilent Feature Extraction Software. To identify up or down-regulated genes, we calculated ratios (non-log scaled fold-changes) from the normalized signal intensities of each probe for comparison between control and experimental samples. We then established criteria for regulated genes: up-regulated genes, ratio ≥2-fold; down-regulated genes, ratio ≤0.5.

### Construct of NDRG1 shRNA and Establishment of NDRG1 Knockdown Cell Lines

To obtain human a U6 siRNA vector based on pcDNA3 (Invitrogen, Carlsbad, CA), the human U6 promoter (containing *Hind*III and *Bam*HI cloning sites) was amplified from human genomic DNA with the primer pair 5′-AGATCTGAATTCCCCAGTGGAAAGACGCGCAGGC and 5′-AGATCTAAGCTTCTCGAGGATCCCGCGTCCTTTCCACAAGATATATAAACCCAAG, and ligated into the *Bgl*II site of pcDNA3. The partial DNA fragments of human NDRG1 DNA were chemically synthesized and cloned into pcDNA3-hU6siRNA cleaved with *Hind*III and *Bam*HI to produce pcDNA3shNDRG1. The synthesized DNAs for pcDNA3shNDRG1 were: 5′-GATCCGCGTGAACCCTTGTGCGGAATTCAAGAGATTCCGCACAAGGGTTCACGTTTTTTGGAAA and: 5′-AGCTTTTCCAAAAAACGTGAACCCTTGTGCGGAATCTCTTGAATTCCGCACAAGGGTTCACGCG. The snail cDNA was prepared as described previously [35]. Snail cDNA was ligated into the pcDNA3 (pcDNA3-Snail). Cells were transfected with pcDNA3shNDRG1 or pcDNA3-Snail using Lipofectamine 2000 (Invitrogen) following the manufacturer’s protocol. Stable transfected clones were established using G418 selection.

### Transfection of Small Interfering RNA

SiRNA corresponding to nucleotide sequences of Snail and β-catenin were purchased from Invitrogen (Carlsbad, CA), respectively, siRNA duplexes were tranfected using Lipofectamine RNAiMAX and Opti-MEM medium (Invitrogen) according to the manufacture’s recommendation.

### Luciferase Assay

To obtain E-cadherin-Luc vector, E-cadhein promoter (−262 to +120) was amplified by PCR using the following primer pairs: 5′- AGATCTTAGTGAGCCACCGGCGGGGC-3′ and 5′- AAGCTTGGCCGGGGACGCCGAGCGAGGG-3′. Underlines indicate restriction enzyme cleavage sites. The amplified fragment was ligated into the pGEM-T easy vector (Promega) and transferred to the pGL3-basic vector (Promega) in BglII and HindIII sites. E-cadherin-luc and pcDNA3-Snail were transfected using Lipofectamine LTX and Opti-MEM medium (Invitrogen) according to the manufacture’s recommendation. After 24 hr, the luciferase activity was measured according to the manufacturer’s instructions (Promega). Furthermore, we also examined luciferase activity driven by β-catenin using TopFlash reporter vector as described previously [18].

### Soft Agar Colony Forming Assay

4×10^3^ cells were plated in 1 ml of culture medium containing 0.36% (w/v) top agar layered over a basal layer of 0.72% (w/v) agar in 6-well plates and allowed to grow for 3–4 weeks. Colonies were photographed and counted in ten random fields of view at 50X magnification using light microscopy. Each experiment was done in triplicate.

### Western Blot Analysis and Fractionation of Nucleus and Cytoplasm

Cells were lysed in buffer containing 50 mM Tris-HCl, 350 mM NaCl, 0.1% NP40, 5 mM EDTA, 50 mM NaF, 1 mM phenylmethylsulfonyl fluoride, 10 µg/mL aprotinin, 10 µg/mL leupeptin, and 1 mM Na3VO4. Total cell lysates were subjected to SDS-PAGE and blotted onto Immobilon membranes (Millipore Corp., Bedford, MA) as described previously [24], [25]. To prepare cytosol and nuclear fraction, cells were lysed in bufferA(10 mM HEPES, pH 7.9, 10 mM KCl, 10 mM EDTA, 1 mM DTT, 0.4% IGEPAL and protease inhibitors) and incubate for 20 min on ice. After centrifugation (3 min, 5,000 rpm), supernatant was used as cytoplasmic fraction. The resulting pellets were resuspended in bufferB (20 mM HEPES, pH 7.9, 200 mM NaCl, 1 mM EDTA, 5% glycerol, 1 mM DTT and protease inhibitors) and incubated on for 2 hr with continuous agitation at 4°C. After centrifugation (5 min, 15,000 rpm), supernatant was used as nuclear fraction. Both nuclear and cytoplasmic fraction were further analyzed by western blotting.

### Quantitative Real-time Polymerase Chain Reaction (qRT-PCR)

Total RNA was isolated from cell culture using ISOGEN reagent (Nippon Gene Co. Ltd., Tokyo, Japan) according to the manufacture’s instructions, as described previously [24], [25]. The primer pairs and probes were obtained from Applied Biosystems. The thermal cycle conditions included maintaining the reactions at 50°C for 2 min and at 95°C for 10 min, and then alternating for 40 cycles between 95°C for 15 s and 60°C for 1 min. The relative gene expression for each sample was determined using the formula 2̂(–delta Ct) = 2̂(Ct(GAPDH)–Ct(target)), which reflected the target gene expression normalized to GAPDH levels.

### Ethics Statement

All animal experiments were approved by the Ethics of Animal Experiments Committee at Kyushu University Graduate School of Medical Sciences. All mice were purchased from Charles River Laboratories and housed in microisolator cages maintained under a 12-hr light/dark cycle. Water and food were supplied *ad libitum*. Animals were observed for signs of tumor growth, activity, feeding, and pain in accordance with the guidelines of the Harvard Medical Area Standing Committee on Animals.

### Evaluation of Tumor Growth

Tumor growth rate was tested by the subcutaneous injection of 1×10^7^ cells suspended in 0.2 mL RPMI1640 medium into 6-week-old female BALB/c nude mice. Mice were examined twice weekly for tumor development. The tumor mass was measured in two dimensions with calipers, and the tumor volume was calculated according to the equation (L×W^2^)/2 (L = length, W-wide).

### Orthotopic and Peritoneal Cavity Implantation

Orthotopic implantation was performed according to Yanagihara *et al* [5]. In brief, 6-week-old female BALB/c nude mice were anesthetized by i.p. injection of 0.28 mg/g 2.2.2-tribromoethanol (Aldrich Chemical, Milwaukee, WI). A small median abdominal incision was made under anesthesia, then 1×10^6^ cells in 0.05-mL volume of RPMI medium were inoculated into the middle wall of the greater curvature of the glandular portion of the stomach using a 30-gauge needle (Nipro, Tokyo, Japan). The stomach was then returned into the peritoneal cavity, and the abdominal wall and skin were closed with an AUTOCLIP applier (Becton-Dickinson, Sparks, MD). The mice were killed when moribund, and peritoneal dissemination was evaluated by counting the number of tumor nodules in the mesenterium. We also assayed for peritoneal dissemination by direct implantation into the peritoneal cavity. We injected 1×10^6^ cells in 0.5-mL volume of RPMI medium into 6-week-old female BALB/c nude mice. On day 55, we calculated the number of tumor nodules on the mesentery and the volume of ascites.

### Immunocytochemical Fluorescence Analysis

Cells were seeded on glass coverslips, and then fixed with precooled methanol (–30°C) for 20 min. After washing with PBS, cells were stained with E-cadherin and β-catenin antibody. The anti- E-cadherin antibody was detected with a Cy3-labeled, anti-mous secondary antibody (Invitrogen). The anti-β-catenin antibody was detected with a 488-labeled, anti-rabbit secondary antibody (Cell Signaling technology). The nuclei were stained with DAPI. The cells were examined with a confocal microscope.

### Immunohistochemical (IHC) Analysis of Xenograft Tumors

IHC analysis of tumors in the xenograft animal model was performed as described previously [24], [25] using specific antibodies against vimentin, E-cadherin, β-catenin and Ki-67.
